# The Interplay between Reproductive Social Stimuli and Adult Olfactory Bulb Neurogenesis

**DOI:** 10.1155/2014/497657

**Published:** 2014-07-22

**Authors:** Paolo Peretto, Roberta Schellino, Silvia De Marchis, Aldo Fasolo

**Affiliations:** ^1^Department of Life Sciences and Systems Biology, University of Turin, Via Accademia Albertina 13 ,10123 Torino, Italy; ^2^Neuroscience Institute Cavalieri Ottolenghi (NICO), Regione Gonzole 10, Orbassano, 10043 Torino, Italy

## Abstract

Adult neurogenesis is a striking form of structural plasticity that adapts the brain to the changing world. Accordingly, new neuron production is involved in cognitive functions, such as memory, learning, and pattern separation. Recent data in rodents indicate a close link between adult neurogenesis and reproductive social behavior. This provides a key to unravel the functional meaning of adult neurogenesis in biological relevant contexts and, in parallel, opens new perspectives to explore the way the brain is processing social stimuli. In this paper we will summarize some of the major achievements on cues and mechanisms modulating adult neurogenesis during social behaviors related to reproduction and possible role/s played by olfactory newborn neurons in this context. We will point out that newborn interneurons in the accessory olfactory bulb (AOB) represent a privileged cellular target for social stimuli that elicit reproductive behaviors and that such cues modulate adult neurogenesis at two different levels increasing both proliferation of neuronal progenitors in the germinative regions and integration of newborn neurons into functional circuits. This dual mechanism provides fresh neurons that can be involved in critical activities for the individual fitness, that is, the processing of social stimuli driving the parental behavior and partner recognition.

## 1. Introduction

Since its rediscovery in the early 90s, the field of adult neurogenesis (AN) has been the object of a large number of studies in order to define its extent and role in the normal brain function, but also to develop new strategies for brain repair. After 30 years of intense research, although the potential use of AN as a ready-made tool in therapy remains elusive (see, for discussion, http://www.frontiersin.org/neurogenesis/researchtopics/adult_neurogenesis_twenty_year/1785 and [1]), its role on brain physiology appears astonishing in the context of adult neural plasticity. AN seems crucial to optimize the plastic responses of the brain in multiple sensory contexts [2]. This important function is achieved through a continuous supply of new neurons into the circuits of the olfactory bulb (OB) and dentate gyrus (DG) of hippocampus, two key regions exerting an important control on brain circuits critical for survival of individuals and species [3, 4].

Newborn neurons show unique functional properties, such as lower threshold for synaptic and structural plasticity, and increased responsiveness to experienced stimuli [5–7], supporting a role for AN in mechanisms of memorization and learning [8]. In addition, recent evidences indicate that integration of “young and excitable” neurons in the DG and OB improves the pattern separation, namely, the ability to distinguish/discriminate between similar/overlapping sensory experiences [2, 9–12]. On the whole, AN is considered as a life-long mechanism, which adapts the brain circuits of each individual according to the environmental complexity and novelty, following a simple rule: more experiences need more cognitive challenges requiring plasticity and in turn more AN [13]. Addition of newborn neurons in the adult brain is orchestrated by a complex interplay between internal and external environmental cues that can either positively or negatively influence different aspects of the neurogenic process (e.g., proliferation of progenitors, migration, differentiation, and survival of neuroblasts) beyond its baseline, which is maintained by an intrinsic control [14–16]. Recently, it has been shown that salient cues such as pheromones released during intersexual and parent-offspring interaction, or pregnancy and postpregnancy hormones, regulate AN [17], thus suggesting a role for AN in the reproductive function (see also [18] for recent discussion). Many questions remain open, as the molecular nature of the cues and the brain circuits underlying the link between reproductive behavior and AN. In this paper, we emphasize that the identification of such players besides tackling the study of AN in biologically relevant contexts gives the chance to investigate how sensory stimuli promoting reproductive behavior are processed in the adult brain. In addition, it also offers a key to understand how the occurrence of AN in two restricted brain regions can impact the whole brain function. Hence, we will briefly describe recent findings linking reproductive social stimuli to AN. Then, we will focus on the accessory olfactory bulb of adult female mice [12, 19] to discuss the role of AN as a mediator of a striking example of sexual behavior, the mate pheromonal imprinting to avoid pregnancy block in female mice [20].

## 2. Adult Neurogenesis and Reproductive Social Stimuli: Some Major Key Points

Physiological modulation of AN has been described in multiple reproductive social behaviors (see for review [17, 21]). The emergence of such behavioral aspects implies multisensory integration with the endocrine system [3, 22, 23].

Pheromonal cues, which convey information about species specificity, gender, social status, health, genetic advantage, and individual recognition [24], have been demonstrated as major stimuli to enhance neurogenesis in the adult brain [12, 19, 25–30]. A direct contact (fully exploration of pheromonal cues and/or coupling) between individuals or with the stimulus (bedding or urine) is necessary to modulate AN [12, 19, 25–40]. Thus, activation of both the main and the accessory olfactory systems, which cooperate in the control of reproductive behavior [41, 42], is required to enhance AN in this context. The relative contribution of the two olfactory pathways on the neurogenic process triggered by olfactory social stimuli remains however poorly understood.

Exposure to semiochemicals, such as those released during sex and parent-offspring interactions, elicits neurogenesis in both SVZ and DG niches [26, 28]. By contrast, exposure to “generic odorants,” or physical activity and environmental enrichment, selectively regulates AN in the SVZ or in the DG, respectively [43, 44]. These data indicate that only salient chemosensory stimuli can trigger neurogenesis simultaneously in both SVZ and DG, possibly through a reciprocal interaction between the two neurogenic niches (see [45] for discussion regarding this point).

AN is also modulated during pregnancy or pseudopregnancy, lactation [32, 46], and in pacing behavior [38, 40], activities that require high-level integration among different brain regions and the endocrine system. The anterior pituitary hormone prolactin (PRL) has been identified as one of the main endogenous cues promoting proliferation of SVZ progenitors in the reproductive context [26–28, 32, 46], suggesting a cooperation between hormones and brain plasticity to generate proper physiological and behavioral responses to the external stimuli [23]. PRL in female rodents rises during mating [47], pregnancy and lactation [48], and after prolonged exposure to male pheromones [27]. Socially induced release of PRL increases proliferation of SVZ progenitor cells, raising the number of newborn neurons integrating into the OB circuits of female mice 15–20 days thereafter. Such an increase is matching with the postpartum period [26, 27, 32]. Hence, PRL-induced proliferation occurring in early gestation is possibly required later on for the expression of normal maternal behaviors during the postpartum period (e.g., pup recognition). It is to note that in the postpartum period, which is characterized by profound hormonal changes [49], cell proliferation in the DG of female rats is reduced [50] and in the SVZ of mice is marked by peculiar fluctuations [32]. Interestingly enough, PRL increases neurogenesis also in male mice during interaction with their own pups, both in SVZ and in DG of hippocampus [28]. Considering the multifaceted role of this hormone in favoring the parental care behavior [48], the PRL-induced neurogenesis might be regarded as a mechanism of neural plasticity eliciting offspring survival [46], although X-ray irradiation of SVZ [36] and genetically targeted ablation of newborn neurons [39] failed to show significant alteration of such behavior.

Exposure to dominant male semiochemicals, besides increasing SVZ neurogenesis through PRL release, enhances cell proliferation in the DG of females via the luteinizing hormone (LH), another hypothalamic-pituitary axis hormone [26]. LH is a well-known mediator of social and reproductive functions [51]. Mak and colleagues [26] correlated such hormone-mediated neurogenic effect with the mate-choice behavior, since only exposure to male dominant chemosensory cues was able to enhance neurogenesis. In parallel, mate preference was lost in females with impaired PRL and/or LH function. By large, these data indicate that AN is part of the complex feedback loops linking pheromones, hormones, and reproductive function [52, 53]. According to this view, social and reproductive cues stimulate steroids hormones secretion and a vast literature shows the influence of gonadal hormones on hippocampal neurogenesis (see for review [54]). By contrast, only a few studies have addressed the role of sex steroids in modulating AN in the OB region [25, 55–57]. This is surprising considering the key role played by estradiol in the control of olfactory reproductive behavior in female rodents [58]. Interestingly, Veyrac and Bakker [57] employing aromatase-knockout mice, which are unable to produce estradiol across their life span, demonstrated that estradiol exposure does not influence SVZ progenitor proliferation but rather differentially affects the survival of newborn neurons and their functional responses to male urine cues in the main and accessory OB. Whether the activity of the estradiol is direct or mediated by other hormones/factors deserves further investigation. Similarly, it remains to be clarified which mechanism selectively improves the estradiol-dependent survival of newborn neurons and their responsiveness to chemosensory stimuli in the main and accessory OB.

Besides hormones, other signals/systems participate in the regulation of AN in the social context of reproduction. Pacing behavior is another interesting example of social interaction which modulates AN in the OB region of rats [38, 40]. Pacing behavior in female rats consists of the possibility to control the rate of sexual stimulation they receive [59], a condition that normally occurs when they mate in seminatural or natural conditions [60, 61]. Paced mating induces several physiological and behavioral advantages, such as a reward state that assures that the behavior will be repeated in the future [62, 63]. Using ovariectomized females, hormonally primed to induce sexual behavior, Paredes and colleagues showed that pacing behavior promotes* per se* an increase in SVZ proliferation that leads to a higher density of new neurons in the accessory OB [38]. In addition, they found that if the behavior is repeated, this increase involves also the main OB. The effect on AN seems specifically dependent on the ability of controlling the sexual interaction and not due to different levels of estradiol and progesterone, since all the different groups had the same hormone and behavioral levels [63]. Pace mating also enhances SVZ-proliferation in males [40], whose sexual activity with receptive females also increases hippocampal proliferation [37]. The authors suggest that increased neurogenesis during pacing behavior could be related to the rewarding value of the sexual interaction and indicate the opioid system as potential modulator of this effect. Accordingly, administration of the opioid antagonist naloxone blocks the rewarding effect induced by sexual behavior in males and females [64, 65] and the opioids modulate AN in the adult brain [66].

In summary, the emerging picture shows that modulation of AN during reproductive social behaviors occurs through multiple exogenous and endogenous cues. These include salient chemosensory signals, adenohypophyseal hormones (e.g., PRL and LH), gonadal hormones, and, potentially, neurotransmitters related to the rewarding value of the sexual interaction. Reproductive social stimuli seem to modulate AN by increasing the proliferation of progenitor cells in both neurogenic niches. In addition, as discussed in detail in the next paragraphs, these stimuli also favor the survival/integration of newborn neurons during a critical time-window of their maturation [12, 19, 30]. This prosurvival effect on newborn neurons is mostly mediated by male pheromonal cues and prominent in the AOB of female mice [11, 19, 30]. From now on, by exploiting the mate pheromonal imprinting in female mice as a specific sex behavior and the process of AN occurring in the AOB of rodents, we will focus on a remarkable example of the interplay occurring between AN and reproductive activity.

## 3. The Mate Pheromonal Imprinting: A Vomeronasal-Dependent Sex Behavior of Female Mice

**Figure 1:**
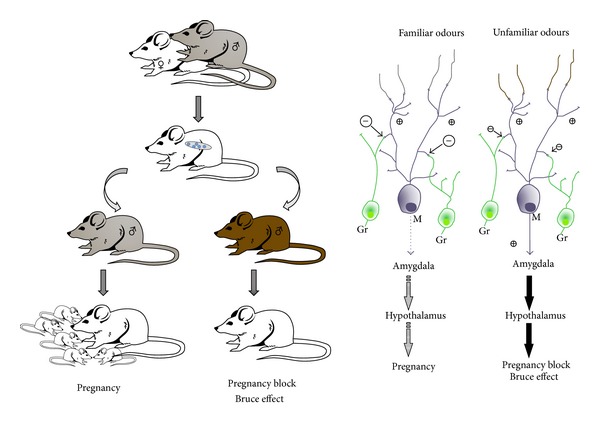
The mate pheromonal imprinting is a vomeronasal-dependent sex behavior. When a recently mated female is exposed to an unfamiliar male (brown mice) or unfamiliar male urine, a neuroendocrine reflex leads to pregnancy block and in turn triggers a return to estrus. This male chemosensory-induced block of pregnancy is known as the Bruce effect (see the text for details). The Bruce effect does not occur if the female is exposed to the mate's chemosignals, since in this case a restricted pool of AOB granule interneurons (Gr) efficiently inhibits mitral cell signal transmission to the forebrain nuclei involved in estrus induction. Such kind of “protection” to the mate's pheromones implies the formation of an olfactory recognition memory for the stud male chemosensory cues at the granule-to-mitral synaptic interface in the AOB (right). Gr: granule cells; M: mitral cells (figure modified from [16]).

The mate pheromonal imprinting is an olfactory recognition process for mate's pheromones, which prevents the pregnancy block effect (also known as the Bruce effect) that occurs during a critical postmating window in adult female mice [67] (Figure 1). If a recently mated female is exposed to chemosignals contained in urine from an unfamiliar male, a neuroendocrine reflex leads to the block of pregnancy and in turn triggers a return to estrus [68, 69]. The Bruce effect is mediated by VN excitatory projections from the AOB to the medial amygdala, the bed nucleus of the stria terminalis, the medial hypothalamus, and ultimately the dopaminergic neurons of the arcuate nucleus that control PRL release by the anterior pituitary [70]. PRL in mice is luteotrophic and its inhibition, mediated by the dopaminergic neurons of the arcuate nucleus, prevents blastocyst implantation [71]. The exteroceptive induction of estrus is lost by stud-male odours through enhancement of granule-to-mitral synaptic inhibition occurring in the AOB during a sensitive period around mating [72, 73] (Figure 1). This process involves a restricted pool of granule cells in the AOB, which actually inhibits for several weeks (50–60 days) mitral cell signal transmission to the forebrain areas involved in estrus induction [72, 73]. Thus, although some of the molecular mechanisms underlying the mate pheromonal imprinting remain to be elucidated (see for detailed discussion [71]) and recent data suggest a possible involvement of some main OB neurons [74], more of four decades of intense investigation supports the idea that such memory process is elicited by male pheromonal cues and mainly targets the vomeronasal system.

Recently, we have proposed that AN in the AOB of female mice plays a critical role in the achievement of this memory process [12], which implies a direct link with reproductive behavior. In the next paragraphs we will discuss multiple experimental evidences supporting this hypothesis.

## 4. The AOB Is a Site of Adult Constitutive Neurogenesis in Rodents

**Figure 2:**
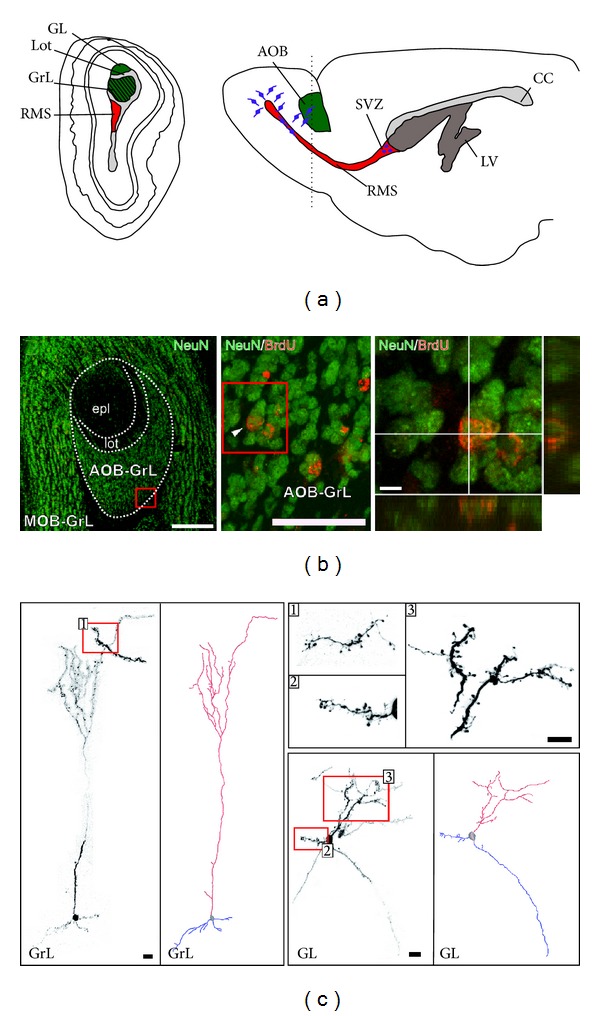
The accessory olfactory bulb is a site of adult constitutive neurogenesis. (a) Left, schematic representation of a posterior coronal section in the rat OB (cut at the level indicated by the dotted line) showing the anatomical organization of the AOB (green) and the RMS (red) in the core of the OB. Right, schematic representation of a parasagittal section of the rodent forebrain showing the position of the AOB (green) and the SVZ neurogenic niche from which neuroblasts migrate to both the MOB and AOB. (b) Coronal cross-section of the OB showing NeuN labeling in the MOB and AOB-GrL. At higher magnification, examples of BrdU/NeuN colabeled cells (see arrowhead) stained 15 d after the BrdU injection. Scale bars, left 200 *μ*m, middle 50 *μ*m, right 5 *μ*m. (c) 3D reconstruction of EGFP-positive SVZ-derived precursors at 60 days after their homotopic transplantation in a wild type mouse [19]. EGFP-positive cells were found in the GrL and GL AOB layers. All cells show features of mature interneurons with well-developed dendritic arborization and spines, as visible at higher magnification. Scale bars, 100 *μ*m and 10 *μ*m (for higher magnification pictures). AOB: accessory olfactory bulb; MOB: main olfactory bulb; SVZ: subventricular zone; RMS: rostral migratory stream; LV: lateral ventricle; CC: corpus callosum; GrL: granular layer; lot: lateral olfactory tract; epl: external plexiform layer; GL: glomerular layer. ((b) was modified from [12] and (c) was modified from [19]).

We reported the presence of SVZ neuroblasts in the AOB (Figure 2(a)) of rats in 1997 [75]. Afterward, other studies from our [76] and other laboratories [31, 77] showed occurrence of immature neurons in the AOB of diverse mammalian species. Yet, clear evidence that SVZ neuroblasts do integrate within the mature circuits of the mouse AOB was obtained only later, when, by using multiple approaches, it was definitively demonstrated that neuroblasts reaching this region acquire phenotypic and anatomical features consistent with those of functional neurons, mostly granule cells (Figures 2(b) and 2(c)), [19]. Subsequent studies have confirmed this evidence [12, 29, 30], which indicates that the AOB, just as the MOB and hippocampus, represents a site of adult constitutive neurogenesis (see for detailed discussion [78]). Since newborn neurons in the adult brain are an elective cellular substrate for mechanisms of memory formation [79], the hypothesis was made that AN in the AOB could serve for the mate pheromonal imprinting.

## 5. Male Bedding Pheromones Modulate Neurogenesis in the AOB of Female Mice

Sensory activity increases integration/survival of newborn neurons in the main OB during a critical time window of neuroblasts maturation [80–82]. Consistent with this “rule,” long- (28 days) or short-term exposure (7 days) to male soiled bedding, which contains semiochemicals present in urine and exocrine glands secretion [83], significantly increases the number of new neurons in the AOB granule cell layer of adult females [12, 19, 29, 30]. This effect is achieved favoring the integration of newborn cells aged between 7 and 14 days [12, 30], the critical time window for survival of AOB newborn neurons [19]. In addition, male bedding exposure can also increase AOB neurogenesis in females by promoting proliferation of SVZ progenitor cells [29]. Enhanced survival in the AOB occurs only in postpubertal females, whereas exposure to cues from both genders is ineffective in both adult males and prepubertal females [12]. These latter data indicate the effect of male pheromones on AN is gender specific and directed only to sexually mature animals.

## 6. Nature of the Cues Affecting AOB Neurogenesis during Male Bedding Exploration

Male bedding is enriched of urine, whose fractions (high- and low-molecular weight fractions; HMW, LMW) are known to contain cues able to elicit different olfactory-mediated reproductive behaviors [84]. Whole urine, as well as the urine fraction deprived of the major urinary proteins (MUPs) that represent principal components of the HMW urine fraction [83], increases AOB neurogenesis in female mice. In addition, the HMW fraction of male urine, treated with menadion to deprive it from all volatile ligands [85] and loaded onto female LMW urine fraction to stimulate investigation is ineffective on AOB neurogenesis [12]. These data strongly support that the cues affecting AN in the AOB of females during bedding exploration are comprised in the LMW fraction of male urine. It is to note that, although MUPs and their bound ligands play a crucial role in providing information in mouse territorial behavior and male individuality [86], the LMW fraction of urine is the most efficient in inducing the Bruce effect [69, 87].

## 7. Circuits Involved in the Regulation of AOB Neurogenesis

Chemosensory stimuli contained in the LMW fraction of male urine are sensed by both the main and the accessory olfactory systems [20]. To evaluate which sensory pathway drives enhanced AOB neurogenesis in females, the effects of genetic deletion of the Trpc2 cation channel, which leads to impaired VN function [88], were compared with those elicited by lesions of the main olfactory epithelium (MOE) caused by intranasal irrigation of zinc-sulfate (ZnSO_4_) [89]. Enhanced AOB granule cell survival was absent in* trpc2* ^−/−^ mice. By contrast, ZnSO_4_ lesion of the MOE did not abolish enhanced neuronal survival in the AOB, indicating that vomeronasal contact is necessary and sufficient to increase survival of new AOB granule cells [12]. This fact is in agreement with previous results showing that AN in the AOB is promoted only by direct contact with male soiled bedding (which implies vomeronasal activity) and not by its volatile compounds [19].

Cellular activity in the AOB can also be elicited by centrifugal inputs from the medial amygdala (MeA), even in absence of vomeronasal stimulation [90, 91]. Convergent olfactory and vomeronasal information reach the amygdala, which actually represents a key center for associative learning of male chemosensory cues [92]. Thus, male bedding exposure was performed after excitotoxic lesions to the MeA by injections of ibotenic acid [93]. Increased cell survival was detected in the AOB of sham-lesioned mice, but not in lesioned ones. Notably, granule cell survival in the main OB was unaffected by either bedding exposure or lesioning with ibotenic acid [12]. Together these results indicate that male pheromones specifically trigger integration of new AOB granule cells through VN centripetal and MeA centrifugal sensory activity. Other studies have shown that AN in the OB is regulated by concurrent peripheral sensory inputs and backward stimulation from central nuclei [94, 95]. In such context, besides central stimuli from MeA, the important noradrenergic input reaching the OB from the locus coeruleus appears intriguing. Indeed, the noradrenergic signaling enhances survival/integration of newborn neurons in both main and accessory OB [30, 96]. In parallel, it also sustains a form of olfactory perceptual learning in which previous experiences allow discrimination between perceptually similar odorants [97], thus supporting that contextual information rather than simple sensory peripheral activity is crucial to enhance the integration/survival of newborn neurons. Finally, the memory formation underlying the mate pheromonal imprinting is dependent on noradrenergic transmission in the AOB [71].

## 8. Functional Involvement of AOB Neurogenesis in the Mate Pheromonal Imprinting

**Figure 3:**
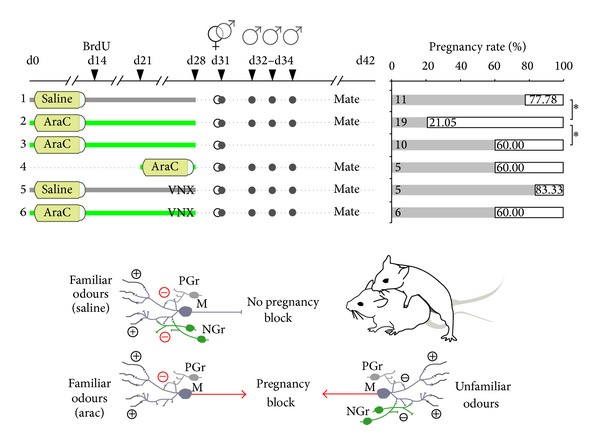
Functional involvement of AOB newborn neurons in the mate pheromonal imprinting. Mating and male olfactory stimulation (physical contact without intromission) on female mice after different protocols of Ara-C/saline treatment in normal and vomeronasal nerve-lesioned mice (VNX). In the graph the pregnancy rates are shown (in percentage) as a function of the different treatment conditions evaluated 11 days after mating. In the schematic diagram the role of AOB newborn granule cells is illustrated (NGr) in the modulation of mate's familiar signals (left side) and unfamiliar ones (right side): granule cells are preferentially involved in the detection of male individual odours once integrated into preexisting circuits. When highly responsive newborn granule cells (NGr) are eliminated after Ara-C treatment (left side, bottom), preexisting granule cells (PGr) are not sufficient to prevent pregnancy block by mate's familiar odours (red arrows). *P < 0.05. (Figure modified from [12]).

Analysis of c-fos expression, as a marker of cellular activity [98], showed preferential responsiveness of AOB newborn neurons to male familiar (1-week experience) rather than unfamiliar (never experienced) stimuli [12]. This effect is evident soon after newborn cell integrates within the AOB circuits (around the 3rd week after their genesis) and is transient since it disappears after seven days. In addition, the same familiar cues induce attenuated responses (low c-fos expression) in the vomeronasal nuclei involved in the control of estrus induction [12]. The above results support direct involvement of AOB newborn cells in male-individual recognition and are consistent with the idea that during a critical time-window of maturation newborn neurons can play unique sensory processing in the OB circuits [5, 6]. Moreover, these data also suggest that the activity of newborn cells in the AOB circuits can modulate the responses of the central nuclei underlying the neuroendocrine reflex leading to the Bruce effect, which actually triggers a return to estrus. To test this hypothesis, the renewal of adult-born interneurons was blocked by administrating the antimitotic drug Ara-C during four weeks (the period of time covering the peak responsiveness to familiar stimuli of AOB newborn neurons). Other methods to ablate AN are available, such as X-ray irradiation [36] or gene-coded selective deletion [39]. Nevertheless, these procedures only partially delete SVZ neurogenesis [36] or require the use of tamoxifen [39], which can potentially interfere with estrogen-responsive regions, such as the vomeronasal system and adult SVZ [99, 100]. By contrast, Ara-C infusion is particularly efficient to eliminate SVZ newborn neurons even after short-term treatments [101] and low doses of Ara-C have no gross aversive side effects [96, 102]. Ara-C treated females were tested for the ability to recognize their mating partners in order to avoid the exteroceptive implantation failure. This was achieved by exposing Ara-C treated females during the postmating critical period (3 days after the beginning of mating in coincidence with the prolactin peaks [69, 87]) to their mating partners (Figure 3). In contrast to saline treated females, high rate of pregnancy failure was detected in Ara-C treated mice, indicating the treatment switched the effect of familiar odour to that of an unfamiliar one. This was not due to a possible Ara-C induced infertility, since a group of Ara-C mated females not exposed during the critical postmating window showed normal pregnancy rate. Thus, ablation of bulbar neurogenesis compromises the formation of the stud male olfactory memory in female mice. To definitively rule out a potential involvement of MOB newborn neurons in this memory formation, a group of Ara-C treated females was tested for the mate pheromonal imprinting after surgical lesion of the vomeronasal nerves, a condition known to eliminate alone the exteroceptive pregnancy block [103, 104]. This procedure was sufficient to prevent the high rate of pregnancy block by stud male exposure [12], showing the key role of AOB newborn interneurons in this process and thus a direct link between AN and reproductive behavior. Importantly, other studies based on depletion of AN obtained through genetic targeting of newborn neurons in the forebrain [39] and/or focal irradiation of the SVZ [36] further support that continuous OB neurogenesis is required to sustain appropriate sex-specific behaviors.

## 9. Concluding Remarks

Social stimuli involved in the control of reproductive physiology enhance AN through complex mechanisms which imply multimodal integration and coordinated activity of the brain with the endocrine system. In turn, newborn neurons seem to optimize/adapt the function of brain circuits underlying reproductive social behaviors. This feedback loop between AN and reproduction further confirms the role of adult neural plasticity in controlling this crucial physiological function and indicates that AN is a key actor of such regulation.

Additional analyses are required to clarify the cellular and molecular mechanisms by which already known external and internal cues modulate AN in reproductive behavior and to test the putative modulatory role on AN of other salient chemosensory factors, such as the exocrine gland-secreting peptide (ESP) 1 [105]. Additional work is needed also in order to enlighten the neural circuits which link AN and reproductive behavior. The vomeronasal pathway and some of its nuclei (e.g., medial amygdala) are important players in this process, but the extensive impact of reproductive social stimuli on different brain regions suggests that other circuits might be involved.

**Figure 4:**
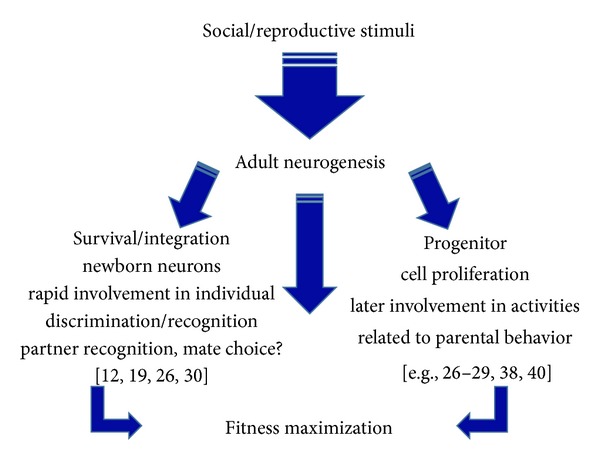
The double effect of social/reproductive stimuli on adult neurogenesis. Reproductive social stimuli positively influence adult neurogenesis by increasing the rate of proliferation of progenitor cells located in the adult germinative niches. This effect, which appears mediated by hormones and/or neurotransmitters released during intersexual and/or parent-offspring interaction, provides a reservoir of newborn neurons that after 2 weeks can be potentially involved in the regulation of parental behaviors. In parallel, socially relevant chemical cues (such as those conveyed by urine) also affect the integration/survival of newborn neurons already settled in the target region (e.g., AOB) during their critical time-window of selection. These additional newborn neurons are rapidly involved in mechanisms of individual discrimination/recognition and play a role in the mate pheromonal imprinting in the AOB of female mice, which is important to avoid pregnancy block. Thus, the positive effect exerted by reproductive social stimuli on AN seems directed to maximize the animal fitness.

Finally, we would like to emphasize that regulation of AN by reproductive social cues in the OB occurs through a dual mechanism: (i) enhancing proliferation of progenitor cells in the SVZ and (ii) fostering survival of newborn neurons in the AOB. Interestingly, in the hippocampus, acute running induces progenitor cell proliferation, but this effect does not lead to a corresponding net increase in neurogenesis if it is not associated with a prosurvival stimulus (i.e., environmental enrichment) [16, 106]. Similarly, the increase of SVZ progenitor proliferation primed by male chemosensory stimuli through hormones release (e.g., PRL) could become “functional” only if an additional appropriate prosurvival stimulus (e.g., chemosensory cues released by pups) eventually enhances newborn cell integration in the OB. By this additive effect, neurogenesis could optimize its function in specific behaviors related to the parental care [27, 28, 46]. In addition to the control on SVZ proliferation, the male chemosensory stimuli also act in parallel by enhancing survival/integration of pools of young newborn neurons in the OB [19, 30]. These cells soon after, or during their integration into the circuits, are highly responsive to experienced/familiar male chemosensory cues, supporting that they can be directly involved in mechanisms of individual recognition/discrimination (Figure 4). Although the logic of this process deserves further investigation, it provides evidence that AN contributes to the reproductive function by affecting key activities related to the animal fitness.
